# What Ancient Egyptian Medicine Can Teach Us

**DOI:** 10.1200/GO.23.00146

**Published:** 2023-06-22

**Authors:** Khaled Elsayad

**Affiliations:** ^1^ Department of Radiation Oncology, University Hospital Muenster, Muenster, Germany

## Abstract

Dr Elsayad describes his impressions regarding the medical and surgical procedures used for patients with cancer and reports on the oncologic cases from ancient Egyptian remains published in the literature.

As a German radiation oncologist who grew up in Egypt and had been working at a university hospital in Germany for 12 years, I decided to stay in my homeland with my family for a year to get closer to the ancient Egyptian civilization. During my stay, I took the opportunity to learn about the history of Egyptian medicine.

Ancient Egyptian medicine is believed to be the origin of Coptic and Greek knowledge, serving as a precursor to science-based medicine. Surviving papers from that time prove the ancient Egyptians' scientific understanding of cancer medicine and their methodical and rigorous process of discovery (Table 1).

**TABLE 1:**
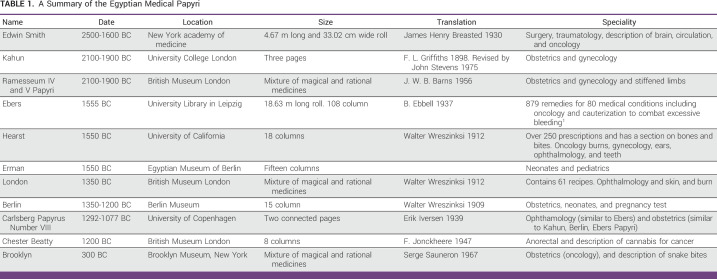
A Summary of the Egyptian Medical Papyri

I am fascinated by the principles and doctrines that were taught in their medical schools, some of which continue to be used today.^1^ What surprised me the most was the active participation of women in medicine, the most prominent among them was Peseshet, the director of lady physicians.^1^ Specialized physicians would take a medical history, perform a manual examination, and assess clinical/vital findings to diagnose a disease.^1^ The range of conditions treated and the various conservative and surgical treatments used are astounding.

Ancient Egyptians understood the importance of maintaining good hygiene and eating habits for good health. The emphasis on healthy food, sport, personal hygiene, daily body cleansing, and mouthwash is still relevant today. I was intrigued to learn that music therapy and amulets were often used to ward off diseases.^2^

Some of the techniques they developed still form the basis of modern medical practices today. One of the fascinating aspects of their medical practices was using sleep therapy with opium or mandrake plant extract to treat patients. Ancient Egyptians had an understanding of sedation techniques even before the development of modern anesthesia.^1^

The way they approached malignancies is also impressive. They could describe primary and metastatic tumors with great detail and recommended removing the entire mass without leaving any gross residual lesions to prevent local recurrence or distant metastasis.

Ancient Egyptian practitioners used cauterization (firing bolt) to treat inflammation and promote healing after surgery. They also described the importance of antiseptic measures even before the development of modern antibiotics, such as using oils and lipids combined with wound dressings to promote postsurgical epithelialization.^3^ It is also interesting to see how advanced their neurosurgical specialty was as they recognized the relationship between brain lesions and the resulting peripheral deficits. This shows that they had a deep understanding of the human nervous system even before the development of modern neuroscience. The discovery of big artificial toes on the feet of numerous mummies is proof for the earliest known functioning prosthetic body organs to date, another remarkable contribution to the life of amputees.^4^

Ancient Egyptians identified natural substances with antibiotic properties, such as honey and onions, that could be used to treat infections.^5^ In addition, they discovered the anti-inflammatory properties of incense, which was derived from the olibanum tree. The Ebers Papyrus describes the use of various plant-based remedies for tumors and found that boswellic acid acetate had antiproliferative effects on malignant cells.^6^ The Egyptians also used natural substances such as the salicin extracted from the willow tree, which was used to reduce inflammation and pain.^7^

Similarly, the shepen plant and propolis were used as a pain killer and sedative for fever and pain.^8^ In addition, carob tree extracts were widely used; this extract contains gallic acid and polyphenol derivatives, which have antimicrobial, antioxidant, and antidepressant effects.^9^ Furthermore, ancient Egyptians used moldy bread on infected wounds thousands of years before Alexander Fleming accidentally discovered penicillin secreted by molds.

Sunlight exposure was used in ancient Egypt to treat various skin disorders and topical remedies to help people with cosmetic concerns.^10^ For example, they used castor oil to promote hair growth in patients suffering from hair loss and cutaneous malignancies associated with alopecia.^11^ They even had an ointment to restore wrinkles and spots, a mixture of honey, red natron, lower Egyptian salt, and alabaster flour.

## Ancient Egyptian Remains and Neoplasms

Studying the remains of ancient Egyptians has always fascinated researchers and scholars alike. As I look at mummies in Egyptian museums all over the world, I wonder about the living conditions, what kinds of substances these people were exposed to, and how much they can tell us about the past.^2^ Radiologic diagnoses have been made possible, even DNA can be extracted through molecular cloning and entered in the International Mummy Database.^12^ The study of ancient Egyptian remains has revealed some insights into the presence of tumors and neoplasms in the population. Recently, the Egyptian genome reference project^13^ has been initiated by Lübecker Institut für Experimentelle Dermatologie (LIED) genetics and systems biology divisions, Lübeck University from Germany and Mansoura University from Egypt.

Several international groups have examined Egyptian remains from various periods. A population study of 905 individuals with an excellent preservation status between 3200 and 500 BC was examined paleopathologically and radiologically (radiographs or computed tomography) using a mummy analysis data system.^12^ Five malignant tumors (three metastatic carcinoma cases and two plasmacytoma cases) affecting the skeleton with multiple osteolytic lesions or osteoblastic reactions were retrieved. The authors concluded that malignant tumors were present throughout the past 4,000 years, with age- and sex-adjusted frequencies not significantly different from industrial populations.^12^ The numerical rise in tumor frequency in present populations is probably related to the longer life expectancy. Nevertheless, all tumor cases were seen in individuals between 1500 and 500 BC, suggesting lower tumor frequencies in the early Egyptian periods.^12^ Previous studies report tumor frequencies in paleopathologic material from ancient Egypt. Armelagos^14^ described one case in a population including 403 mummies, and El-Rakhawy et al^15^ reported on one case from 222 remains. Moreover, Strouhal^16^ identified multiple cases with carcinoma from hundreds of mummies in 1976 and 1982. Afterward, Rösing^17^ described four cases with a malignancy of 1,180 analyzed mummies. The most common primary tumors in ancient Egypt were nasopharyngeal malignancy,^15^ bone metastases, sarcomas, and plasmacytoma.^12^ In addition, primary rectal carcinoma with sacral infiltration and bony destruction was also demonstrated.^18^ Moreover, two cases with multiple basal cell naevus syndrome in ancient Egyptian remains have been published.^19^

Similar to malignant tumors, various benign neoplasms have been discovered in ancient Egyptian remains. For example, a case of a calcified myoma measuring 12.3 cm × 8 cm × 10.5 cm has been described.^20^ Regarding the musculoskeletal system, an osteoma, a chondroma, and a giant tumor have been demonstrated in several radiologic studies.^15^ In addition, an epidermoid cyst, angioma, cystadenoma, and epithelioma have also been reported.^21^ Interestingly, on the basis of the clinical and radiologic manifestations, a hormonally active tumor of the adrenal gland has been suggested in the case of King Tutankhamun.^22^

As I learn more about the health of ancient Egyptians, I discover that they faced many of the same challenges that we do today. In particular, cancer was present and could be just as devastating as it is now. But what is most striking is how these patients survived for long periods despite their illnesses. In advanced or terminal cases, surgery was declined.^2^ One example, in particular, stands out. El-Rakhawy et al described a male patient who suffered from an advanced and erosive lesion of the right nasopharynx. The floor of the pterygoid fossa had opened into the maxillary sinus, causing severe pain that is suggested by a piece of linen cloth found in the right auditory meatus. Despite this excruciating pain, the patient survived for a considerable period, partly thanks to the help and care of those around him.^15^ It is a testament to the care and support that they must have received from their families and communities. It is also a reminder that medicine has always been about more than just treating the physical symptoms of disease.

We have only a glimpse into the medical knowledge of Ancient Egyptians, but enough to recognize that they used medical and surgical procedures to help improve the clinical outcome for patients with cancer. But beyond that, they had a deep sense of community and compassion for those struggling with illness. It is a reminder that although medicine might have changed over the centuries, the fundamental human need for care and support remains the same.
